# Is MHC diversity a better marker for conservation than neutral genetic diversity? A case study of two contrasting dolphin populations

**DOI:** 10.1002/ece3.5265

**Published:** 2019-05-23

**Authors:** Oliver Manlik, Michael Krützen, Anna M. Kopps, Janet Mann, Lars Bejder, Simon J. Allen, Celine Frère, Richard C. Connor, William B. Sherwin

**Affiliations:** ^1^ Biology Department, College of Science United Arab Emirates University Al Ain United Arab Emirates; ^2^ Evolution and Ecology Research Centre, School of Biological, Earth and Environmental Sciences University of New South Wales Sydney New South Wales Australia; ^3^ Department of Anthropology University of Zurich Zurich Switzerland; ^4^ Department of Biology and Department of Psychology Georgetown University Washington District of Columbia; ^5^ Marine Mammal Research Program, Hawai'i Institute of Marine Biology University of Hawai'i at Manoa Kaneohe Honolulu; ^6^ Aquatic Megafauna Research Unit, School of Veterinary and Life Sciences Murdoch University Murdoch Western Australia Australia; ^7^ School of Biological Sciences University of Bristol Bristol United Kingdom; ^8^ Faculty of Science, Health, Education and Engineering University of the Sunshine Coast Sippy Downs Queensland Australia; ^9^ Biology Department UMASS‐Dartmouth Dartmouth Massachusetts

**Keywords:** adaptive genetic variation, bottlenose dolphin, cetacean, conservation genetics, major histocompatibility complex, microsatellites

## Abstract

Genetic diversity is essential for populations to adapt to changing environments. Measures of genetic diversity are often based on selectively neutral markers, such as microsatellites. Genetic diversity to guide conservation management, however, is better reflected by adaptive markers, including genes of the major histocompatibility complex (MHC). Our aim was to assess MHC and neutral genetic diversity in two contrasting bottlenose dolphin (*Tursiops aduncus*) populations in Western Australia—one apparently viable population with high reproductive output (Shark Bay) and one with lower reproductive output that was forecast to decline (Bunbury). We assessed genetic variation in the two populations by sequencing the MHC class II DQB, which encompasses the functionally important peptide binding regions (PBR). Neutral genetic diversity was assessed by genotyping twenty‐three microsatellite loci.

We confirmed that MHC is an adaptive marker in both populations. Overall, the Shark Bay population exhibited greater MHC diversity than the Bunbury population—for example, it displayed greater MHC nucleotide diversity. In contrast, the difference in microsatellite diversity between the two populations was comparatively low.

Our findings are consistent with the hypothesis that viable populations typically display greater genetic diversity than less viable populations. The results also suggest that MHC variation is more closely associated with population viability than neutral genetic variation. Although the inferences from our findings are limited, because we only compared two populations, our results add to a growing number of studies that highlight the usefulness of MHC as a potentially suitable genetic marker for animal conservation. The Shark Bay population, which carries greater adaptive genetic diversity than the Bunbury population, is thus likely more robust to natural or human‐induced changes to the coastal ecosystem it inhabits.

## 1 INTRODUCTION

A loss of genetic diversity is often associated with reduced fitness and can negatively impact population viability (Chapman, Nakagawa, Coltman, Slates, & Sheldon, 2009; Frankham, Ballou, & Briscoe, 2010; Reed & Frankham, 2003). Until recently, studies that assessed genetic diversity in wild animal populations typically used adaptively neutral genetic markers, such as microsatellites. However, neutral genetic markers offer little insight into the adaptive potential to cope with natural and artificial change (Allendorf & Luikart, 2008; Hedrick, 2005; Holderegger, Kamm, & Gugerli, 2006). Therefore, to assess genetic diversity that captures information relevant to the conservation of populations, it is prudent to use genetic markers linked to ecologically important traits (Manlik, Schmid‐Hempel, & Schmid‐Hempel, 2017; Piertney & Webster, 2010; van Tienderen, Haan, Linden, & Vosman, 2002).

One such adaptive marker is the major histocompatibility complex (MHC) (reviewed by Sommer, 2005). The MHC plays an important role in responding to antigens and initiating an immune response in vertebrates. Major histocompatibility complex variation has been associated with various fitness traits, including factors important for population viability, such as resistance to parasites, survival, and reproductive success (Hedrick, 2003; Kalbe et al., 2009; Kurtz et al., 2004; Sepil, Lachish, Hink, & Shelcon, 2013; Sepil, Lachish, & Sheldon, 2013; Thoss, Ilmonen, Musolf, & Penn, 2011; Wegner, Kalbe, Milinski, & Reusch, 2008). High levels of MHC diversity observed across a variety of vertebrate species are commonly explained by balancing selection (Garrigan & Hedrick, 2003). Balancing selection maintains high levels of MHC diversity by two possible, not mutually exclusive, mechanisms: frequency‐dependent selection (Borghans, Beltman, & Boer, 2004) and heterozygote advantage (Doherty & Zinkernagel, 1975). The frequency‐dependent selection model suggests that MHC diversity is pathogen‐mediated, because rare MHC variants are selected for by host‐pathogen co‐evolution. In contrast, heterozygote advantage explains balancing selection due to heterozygotes having greater fitness than homozygotes.

Compared to terrestrial vertebrates, relatively little is known about MHC diversity in cetaceans, and the extent to which cetacean MHC diversity is associated with population viability remains uncertain. The vaquita (*Phocoena sinus*) population, endemic to the Gulf of California, showed low levels of MHC II variation (Munguia‐Vega et al., 2007) and is now considered functionally extinct (Taylor et al., 2017). In contrast, the extinct baiji (*Lipotes vexillifer*) of the Yangtze River exhibited very high MHC diversity (Xu et al., 2012; Yang, Yan, Zhou, & Wei, 2005). Reduced MHC diversity may not necessarily adversely affect population viability (Radwan, Biedrzycka, & Babik, 2010). Caveats for many of these studies are that they had no baseline measure of genetic diversity in a conspecific viable population or no comparison of MHC and other types of genetic variation. No study to date has compared MHC and neutral genetic diversity of conspecific cetacean populations that differ with respect to population parameters and viability forecasts.

**Figure 1:**
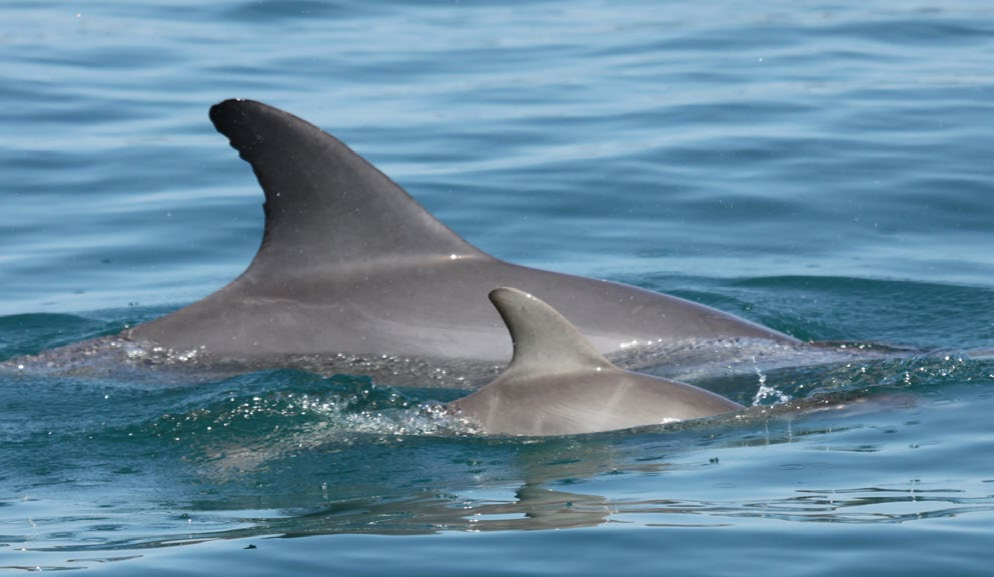
Mother and calf bottlenose dolphin (*Tursiops aduncus*) in Shark Bay, a UNESCO World Heritage Site in Western Australia. Photograph: Ewa Krzyszczyk

**Figure 2:**
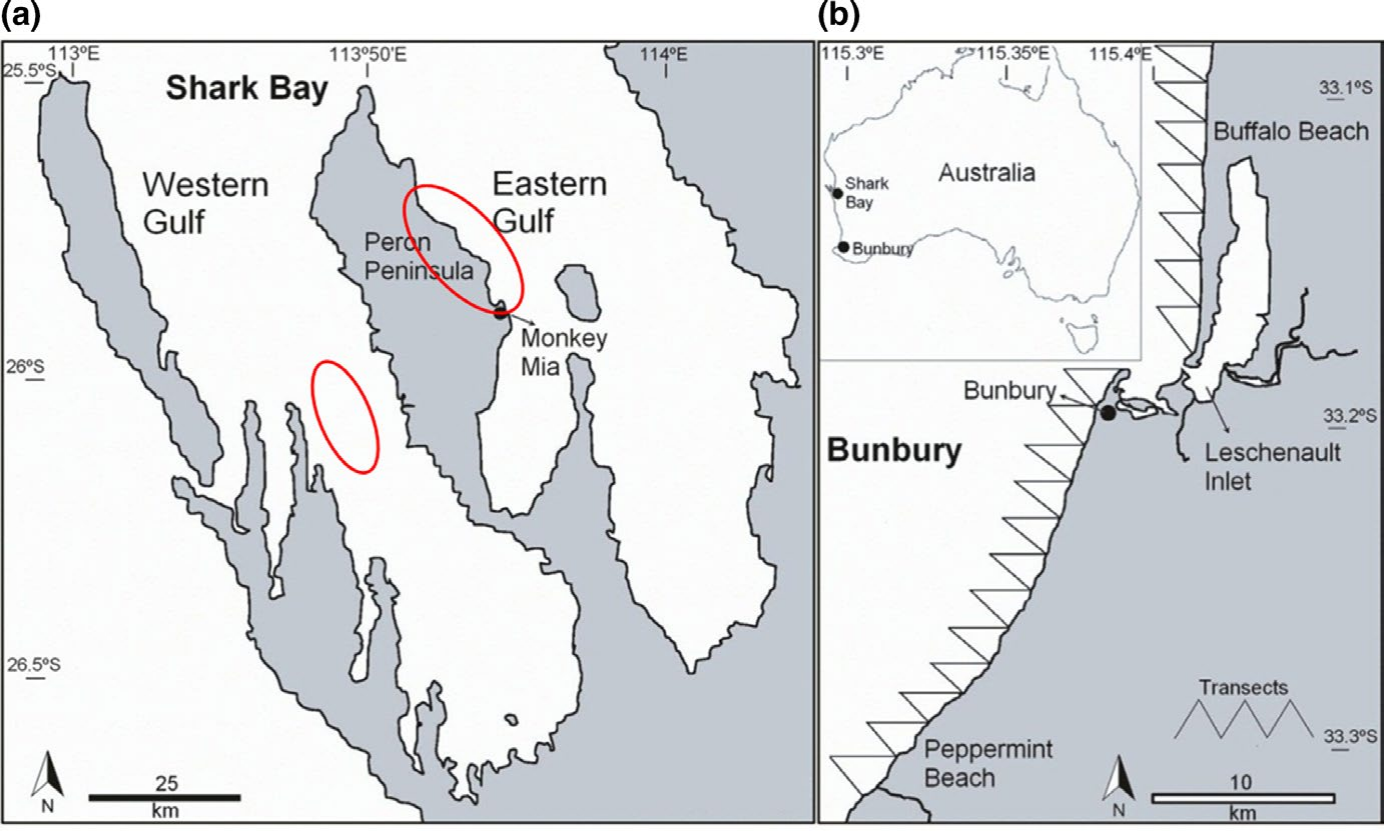
(a) Shark Bay, a UNESCO World Heritage area, is about 13,000 km^2^ in size and is divided by the Peron Peninsula, which bisects the bay into a western and an eastern gulf. Sampling sites included a 300 km^2^ area (circled) north of Monkey Mia and an area of ca. 260 km^2 ^(circled) in the western gulf. (b) The inset shows the relative location of the study sites (Shark Bay & Bunbury). The coastal study area of Bunbury covers about 120 km^2^ and extends approximately 1.5 km offshore with a linear distance of 50 km. The study site includes the coastal areas, embayment, Leschenault Inlet and outer harbors (5 km^2^), estuary and river mouth (15 km^2^). Transects of the outer‐water Bunbury study site are shown. These figures are modified from Figure S1 of Manlik et al. (2016)

In this study, we used two genetic markers, MHC and neutral microsatellites, to assess genetic diversity of two contrasting bottlenose dolphin (*Tursiops aduncus*) populations—one in Shark Bay (SB) and another off Bunbury (BB), Western Australia (Figure 1). These two populations, more than 1,000 km apart (Figure 2), are not connected by dispersal. Each population exhibits limited genetic exchange with its neighboring populations (Allen et al., 2016; Manlik et al., 2018). The two populations differ greatly with respect to population viability. A comparative population viability analysis showed that the SB population appeared stable, but the BB population was forecast to decline with a high probability of extinction, unless supported by immigration (Manlik et al., 2016). The large difference in viability between the two populations was best explained by considerable differences in reproductive rates (Manlik et al., 2016). Besides this difference in reproductive rates, the two populations also differ with respect to anthropogenic pressure (Manlik et al., 2016). The SB population occurs in a remote UNESCO World Heritage area with markedly lower anthropogenic activity, whereas BB inhabits waters adjacent to an expanding regional city and port with comparatively high vessel traffic (Manlik, 2019; Manlik et al., 2016; Nicholson, Bejder, Allen, Krützen, & Pollock, 2012; Smith, Frère, Kobryn, & Bejder, 2016; Sprogis et al., 2018).

SB and BB also differ with respect to reported population sizes. SB population size was estimated by aerial surveys to be about 2,000–3,000 individuals (minimum estimates; Preen, Marsh, Lawler, Prince, & Shepherd, 1997) in a 14,900 km^2^ area, but other studies investigating various sections of SB suggest that the population may be much larger (e.g. Nicholson et al., 2012). BB population size was assessed to be approximately 260 individuals for the 120 km^2^ area (Manlik et al., 2016). However, smaller seasonal abundance estimates have been reported for BB (Smith, Pollock, Waples, Bradley, & Bejder, 2013; Sprogis et al., 2016). Different methodologies to estimate population sizes, and the issue of connectivity, make comparison difficult, but all studies suggest that SB is substantially larger than BB.

The aim of this study was to compare MHC II DQB genetic diversity and microsatellite diversity between these two contrasting dolphin populations. Given that only few MHC studies have been conducted on populations with differing reproductive success or population forecasts, this provided a rare opportunity to compare MHC and neutral genetic diversity between two natural populations with considerable differences in viability. If MHC variation reflects differences in fitness, and given the large difference in reproductive output between the two populations (Manlik et al., 2016), we would expect to observe a larger inter‐population difference in MHC diversity than in microsatellite diversity. Additionally, to assess evolutionary and ecologically relevant genetic variation, we evaluated signals of selective pressure on MHC II DQB. We did this by assessing nonsynonymous versus synonymous nucleotide substitutions (Nei & Gojobori, 1986), whether substitutions occurred at codons expressing antigen‐binding residues, and by performing a Tajima's *D* test (Tajima, 1989).

## 2 MATERIALS AND METHODS

### 2.1 Sample collection and DNA extraction

Between 1997 and 2013, we opportunistically collected skin samples from free‐ranging bottlenose dolphins in Shark Bay (SB) and off Bunbury (BB), Western Australia (Figure 2), using a biopsy system designed for small cetaceans (Krützen et al., 2002). Tissue samples were stored in a saturated NaCl/20% (v/v) dimethyl sulfoxide solution for DNA stabilization (Amos & Hoelzel, 1991). We isolated genomic DNA following standard phenol–chloroform protocol (Davis, Dibner, & Battey, 1986), or alternatively using the Gentra Puregene Tissue Kit (Qiagen).

Sampling in SB included two sites in western and eastern SB (Figure 2), that are connected by extensive gene flow (Krützen, Sherwin, Berggren, & Gales, 2004) and appear to form one large continuous population. A total of 686 and 125 dolphins were biopsied in SB and off BB, respectively. Sex of individuals was determined by various methods, as described by Sprogis et al. (2016), including genetic sexing (Baker et al., 1998). We performed chi‐square tests to assess whether the numbers of males and females in the samples were significantly different from those in the surveyed populations or different from an expected 50:50 male to female ratio. Sex ratios for surveyed individuals versus sampled individuals were not significantly different (SB = *χ* ^2^ = 0.42, *p* = 0.515; BB: *χ* ^2^ = 0.16, *p* = 0.693) nor were the ratios of sampled individuals significantly different from 50:50 (SB: *χ* ^2^ = 0.10, *p* = 0.757; BB: *χ* ^2^ = 1.7, *p* = 0.190). To assess whether it was justified to pool samples collected from eastern and western SB, we estimated subpopulation fixation index (*F_ST_*) based on microsatellite data, using genalex 6.501 (Peakall & Smouse, 2006, 2012), and compared MHC and microsatellite diversity between the two sampling locations.

Data were collected under research permits (SF005997; SF006538; SF007046; SF007596; SF008480; SF009119) licensed by the Western Australian Department of Environment and Conservation (now the Western Australian Department of Parks and Wildlife). This study was carried out in accordance with the Murdoch University Animal Ethics Committee approval (W2076/07; W2307/10; W2342/10).

### 2.2 Amplification, Sanger sequencing, and sequence variant determination

To characterize MHC genetic variants of the two populations, we amplified and sequenced the MHC II DQB exon 2 (hereafter MHC DQB), which encompasses the functionally important PBR (Baker et al., 2006; Hayashi et al., 2003; Hoelzel, Stephens, & O'Brien, 1999; Murray, Malik, & White, 1995; Seddon & Ellegren, 2002), and which is the region under strongest selection (Hughes & Nei, 1989). Amplification was performed using the universal primer pair DQB1 and DQB2, 5′CATGTGCTACTTCACGTTCGG 3′ (forward), 5′CTGGTAGTTGTGTCTCCACAC 3′ (reverse), which were originally designed by Tsuji, Aizawa, and Sazaki (1992), and previously used to amplify cetacean MHC (Caballero et al., 2010; Du, Zheng, Wu, Zhao, & Wang, 2010; Hayashi et al., 2003, 2006; Heimeier et al., 2009; Moreno‐Santillán, Lacey, Gendron, & Ortega, 2016; Murray et al., 1995; Vassilakos, Natoli, Dahlheim, & Hoelzel, 2009).

PCR for MHC DQB was performed using 1.25 μM primers, 0.2 mM dNTPs, 1.0 mM MgCl_2_, 1.25 U GoTaq DNA polymerase (Promega), and 20–100 ng (5 μl) of template DNA in a total volume of 25 μl. Thermal cycling was conducted on an Eppendorf Mastercycler (ep gradient S) with an initial denaturing temperature of 95°C for 15 min, 30–35 cycles of denaturation at 95°C (1 min), and annealing at 55°C (30 s), followed by an elongation step at 72°C for 1 min.

PCR products were visualized by electrophoresis on a 1.5% agarose gel (1× TBE buffer) stained with GelRed™ (Biotium). All MHC amplicons were sequenced in the forward and reverse direction using Big Dye 3.1 on a 3730xl DNA Analyzer (Applied Bioscience) at the Ramaciotti Centre of the University of New South Wales.

MHC DQB Sanger sequences (172 bp; forward and reverse) were aligned with clustalw (Thompson, Higgins, & Gibson, 1994) in geneious 6.1 (Drummond et al., 2010). Double‐peaks were called using the “Heterozygotes” plugin (geneious) based on the default threshold of 50% peak height and double‐checked by visual inspection. Subsequently, MHC DQB sequence variants were inferred by reconstructing haplotype phases from the unphased sequence alignment data using the coalescent‐based Bayesian method phase (Stephens & Donnelly, 2003; Stephens, Smith, & Donnelly, 2001) in dnasp version 5.10.01 (Librado & Rozas, 2009) with 100 iterations, 1 thinning interval and 100 burn‐in iterations. phase was shown to be reliable for reconstructing haplotypes (Stephens & Donnelly, 2003), including MHC haplotypes (Bos, Copurenko, Williams, & DeWoody, 2008; Bos, Turner, & DeWoody, 2007; Silva & Edwards, 2009). After haplotype reconstruction, the MHC DQB alignments in dnasp contained sequences for 276 SB and 65 BB individuals. We performed a blastn search to compare inferred MHC DQB sequence variants to sequences in the NCBI database.

### 2.3 Assessing signals of selection acting on MHC DQB

To assess signals of selection, we compared rates of nonsynonymous (*d_N_*) and synonymous (*d_S_*) substitutions within the 172‐bp MHC DQB region. We used the Nei–Gojobori method (Nei & Gojobori, 1986) for a codon‐based test of positive selection (two‐sided *z*‐test) implemented in MEGA version 7.08 (Kumar, Stecher, & Tamura, 2016) to test whether *d_N_* > *d_S_* for (a) all codons of the entire sequence; (b) codons of the putative peptide binding region (PBR), that is, variable codons that code for amino acids that have been reported to bind to antigens; and (c) putative nonpeptide binding regions (non‐PBR). Variance estimation for the *z*‐test was based on 1,000 bootstrap replicates. Additionally, we used DNASP to perform Tajima's *D* test (Tajima, 1989), which detects departure from selective neutrality or historical changes in population size.

### 2.4 Assessment of MHC sequence diversity

After alignment in geneious, we compared sequence variation using dnasp 5.10.01 (Librado & Rozas, 2009). We recorded the following measures of sequence variation: (a) nucleotide diversity (*π*), as described by Nei (1987) (equation 10.5), (b) haplotype diversity (*Hd*) (Nei, 1987; equation 8.4), (c) Watterson mutation estimator (*Ө_W_*), according to Watterson (1975) (equation 1.4), and (d) the mutation parameter, theta (*Ө_Eta_*) per nucleotide site from the total number of mutations (Nei, 1987; equation 10.3).

Sampling variances and standard deviations were calculated for nucleotide diversity and haplotype diversity according to Nei (1987) and for Watterson mutation estimator according to Tajima (1993). We also calculated standard errors of the mean between the three SB conservative samples and across all subsamples (SB: 19 subsamples; BB: 5 subsamples). We used *t* tests to compare the mean *π*, *Hd*, *Ө_W_*, and *Ө_Eta_* values between the two populations across all subsamples.

### 2.5 Assessment of microsatellite diversity

All sampled BB individuals were previously genotyped for 25 polymorphic microsatellite loci (Manlik et al., 2018). We followed the same procedure and checks for genotyping individuals of the SB population as described in Manlik et al. (2018): We used previously tested primers for polymorphic microsatellite loci (Hoelzel, Potter, & Best, 1998; Kopps et al., 2014; Krützen, Valsecchi, Connor, & Sherwin, 2001; Nater, Kopps, & Krützen, 2009; Shinohara, Domingo‐Roura, & Takenaka, 1997). All primer sequences used in this study are listed in Dryad/Table S1. Microsatellite amplification was performed using the Qiagen Multiplex Kit^TM^ in three multiplex PCR reactions as described in Manlik et al. (2018). Fragment analysis of PCR amplicons was performed on a 3730XL DNA Analyzer (Applied Biosystems), employing a Genescan‐500 LIZ^TM^ size standard. Alleles were scored using genemapper 4.0 (Applied Biosystems) and the microsatellite plugin for geneious 6.0 (Drummond et al., 2010). We used Micro‐Checker version 2.2.3 (van Oosterhout, Hutchinson, Wills, & Shipley, 2004) to test for scoring errors due to stuttering and the presence of large‐allele dropouts across all loci and populations. The software INEst version 2.0 (Chybicki & Burczyk, 2009) was used to estimate the frequency of null alleles at microsatellite loci in each population. Linkage disequilibrium for all microsatellite locus pairs was tested with genepop version 4.5.1 (Rousset, 2008). We used GenAlEx 6.501 (Peakall & Smouse, 2006, 2012) to test all loci for departures from Hardy–Weinberg equilibrium (HWE).

Microsatellite diversity was summarized by measuring observed heterozygosity (*H_o_*), expected heterozygosity relative to HWE (*H_e_*), the number of effective alleles (*A_e_*), and Shannon's Index (*^1^H*) (Brown & Weir, 1983; Sherwin, Chao, Jost, & Smouse, 2017), using genalex 6.501. We used paired *t* tests to compare the mean values of these measures between the two populations across the microsatellite loci.

### 2.6 Sampling for comparison of inter‐population genetic diversity

Due to the sample‐size difference between SB and BB, we used three sampling approaches to compare genetic diversity between the two populations:

1. Maximum sampling: We sampled the maximum number of individuals for which we obtained MHC DQB sequences or microsatellite genotypes. For SB, this approach included 276 individuals for which we obtained MHC DQB sequences and 667 individuals for which we genotyped for the microsatellite loci. For BB, the maximum sampling included MHC sequences of 65 individuals and microsatellite genotypes of 84 individuals.
2. Conservative sampling: We first reduced the maximum sample set to only include individuals for which we had both MHC DQB and microsatellite data. This resulted in 55 samples for BB and 239 for SB. In order to compare equal sample sizes that reflect the demography of the two populations, we further subsampled the SB data to include the same number of each of the age classes (calves, juveniles, and adults) and sexes that were found in the conservative BB sample (Dryad/Table S2). From the SB sample set that included 239 individuals, we obtained three subsamples (SB samples 1–3), each containing the same numbers for each of the age classes and sexes found in the BB conservative sample. We did this by randomly choosing from the SB samples 2 calves, 15 juveniles, and 38 adults, of which 32 were males and 23 were females. Individuals of unknown age classes or sexes were excluded. Each of the individuals was only sampled once, for example individuals included in SB sample 1 were not included in SB sample 2 or 3.
3. Subsampling: In order to allow for statistical comparisons of MHC diversity measures between SB and BB, we subsampled both populations by randomly choosing 11 samples from each conservative sampling set of each population. Each sample was only included once in each subsample. This generated 19 × 11 (209) subsamples for SB and 5 × 11 (55) subsamples for BB.

Other methods, such as rarefaction, are often used to investigate the effect of sample size, but we believe our three sampling approaches address this more thoroughly.

## 3 RESULTS

Pooling the eastern and western SB datasets was justified because the subpopulation fixation index (*F_ST_*) comparing the two sampling sites in SB showed very little differentiation (*F_ST_* = 0.006; Dryad/Table S3), indicating that the two sites represent one population. Also, MHC and microsatellite diversity of the two SB sampling sites were similar (Dryad/Table S3).

### 3.1 Sequence variants of MHC DQB

Forward and reverse MHC DQB sequences of a total 341 individuals (SB: 276; BB: 65) were analyzed. Totals of 186 and 43 MHC DQB sequence variants were inferred by haplotype reconstruction for SB and BB, respectively. We did not detect any patterns in the sequences that indicated multiple allelism (i.e. having more than two alleles or sequence variants per amplicon/individual), gene duplications, stop codons, or frameshifts. Comparing MHC DQB sequences of seven mother–father–offspring trios (Kopps, 2007) did not reveal any patterns that were inconsistent with single‐locus Mendelian inheritance. Nonetheless, we refrain from classifying these inferred sequence variants as novel MHC alleles, which are commonly confirmed by sequencing clones (Marsh et al., 2010) or by re‐genotyping all individuals with rare haplotypes (Ahmad et al., 2002). Thirty‐nine individuals were homozygous for all 172 bps for one of four unique sequences (Dryad/Figure S1). All sequences showed high similarity (98%–100%) to published MHC DQB alleles in dolphins (Dryad/Table S4).

### 3.2 Signals of selection acting on MHC

We detected signals of selection acting on MHC DQB. In both populations, nonsynonymous (*d_N_*) substitution rates were significantly greater than the synonymous (*d_S_*) substitutions rates in the entire 172‐bp region and in the putative PBR, but not in the non‐PBR (Table 1). About 82% of the variable nucleotide sites (18 out of 22) are within codons that have been associated with the PBR (Dryad/Figure S1). Notably, the large majority of variable nucleotide sites were detected within the PBR (Dryad/Figure S1). Tajima's *D* was near zero for MHC DQB in both populations (Table 2). However, in the SB population it significantly departed from zero (*D* = −1.82, *p* < 0.05) when considering only nonsynonymous substitutions (Table 2).

**Table 1:** The estimated rates of nonsynonymous (*d_N_*) and synonymous (*d_S_*) substitutions (±standard errors of the mean) for putative peptide binding regions (PBR) and nonpeptide binding regions (non‐PBR) and their ratios for DQB exon 2 in the Shark Bay (SB) and Bunbury (BB) dolphin population

Pop.	Sites	*N*	*d_N_*	*d_S_*	*d_N_/d_S_*	*z*	*p*
SB	PBR	17	6.334 ± 1.196	3.974 ± 1.439	1.59	4.563	0.0000061
	Non‐PBR	39	1.539 ± 0.205	1.659 ± 0.446	0.93	0.276	ns
	All	56	3.500 ± 0.558	3.095 ± 0.778	1.13	2.976	0.0018
BB	PBR	17	2.297 ± 0.536	1.362 ± 0.723	1.69	3.033	0.0015
	Non‐PBR	39	0.244 ± 0.0849	0.197 ± 0.134	1.24	1.518	ns
	All	56	0.993 ± 0.224	0.694 ± 0.309	1.43	3.023	0.0015

Note

*N* is the number of codons in each category. The *p*‐value is the significance value for the difference between *d_N_* and *d_S_*, using a two‐sided *z*‐test.

**Table 2:** Results of Tajima's *D* tests performed on all nucleotide sites and nonsynonymous sites within the MHC DQB of the Shark Bay (SB) and Bunbury (BB) dolphin population

MHC DQB region	SB			BB		
	*D*	*p*	Sig.	*D*	*p*	Sig.
All sites	−1.54	0.10 > *p*> 0.05	ns	−0.55	*p* > 0.10	ns
Nonsynonymous sites	−1.82	<0.05	sig.	−0.81	*p* > 0.10	ns

### 3.3 MHC sequence diversity

**Figure 3:**
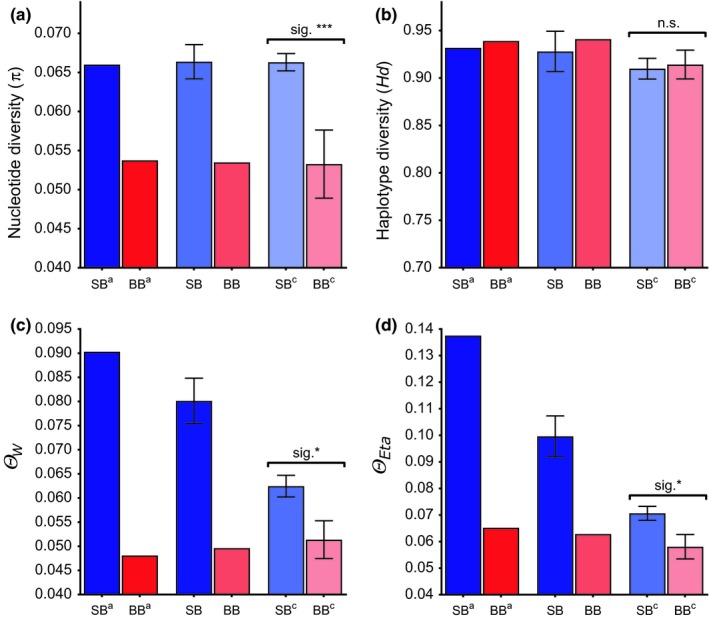
MHC DQB genetic diversity of dolphins in Shark Bay (SB; blue bars) and Bunbury (BB; red bars) (a) nucleotide diversity (*π*), (b) haplotype diversity (*Hd*), (c) Watterson mutation estimator from variable sites (*Ө_W_*), and (d) theta per site from Eta (*Ө_Eta_*). Each pairing of SB‐BB bars represents the results on the basis of each of the three sampling approaches: maximum sampling (SB^a^, BB^a^), conservative sampling (SB^b^, BB^b^), and subsampling (SB^c^, BB^c^). Whiskers depict the respective standard errors of the mean, which are only shown for means across subsamples. Significant values based on *t* tests: sig. *p < 0.05; sig. ***p < 0.0005; n.s. = nonsignificant (*p* > 0.05)

Regardless of the sampling approach (maximum, conservative, or subsampling), dolphins of SB showed greater MHC DQB diversity than those of BB, except with respect to *Hd*, which showed no significant difference (Figure 3; Table 3). On the basis of the conservative sampling approach, mean *π* of the SB population was 0.066 (*SE* = 0.0022), substantially greater than that of BB (*π* = 0.053, *SE* = 0.0018; Figure 3a). In comparison with BB, the SB population showed larger *Ө_W_* (SB: *Ө_W_* = 0.0801, *SE* = 0.0047; BB: *Ө_W_* = 0.0496, *SE* = 0.0029; Figure 3c). Theta (*Ө_Eta_*) was substantially larger for SB (*Ө_Eta_* = 0.100, *SE* = 0.0077) than for BB (*Ө_Eta_* = 0.063, *SE* = 0.0049; Figure 3d). Based on the subsampling approach, *π*, *Ө_W_*, and *Ө_Eta_* were significantly greater for SB than for BB (Figure 3; Table 5).

**Table 3:** MHC II DQB diversity measures based on conservative and maximum sampling approaches

Pop	Sampling approach	*n*	*π*	*Hd*	*Ө_W_*	*Ө_Eta_*	*Eta*
Bunbury	BB max.	65	0.0538 (0.0025)	0.939 (0.013)	0.0481 (0.013)	0.0652	61
	BB cons.	55	0.0535 (0.0025)	0.941 (0.014)	0.0496 (0.014)	0.0629	57
	BB subsampling mean (5 × 11)	11	0.0533 (0.0010)	0.914 (0.015)	0.0514 (0.039)	0.0581	35.2
Shark Bay	SB max.	276	0.0660 (0.0014)	0.932 (0.0084)	0.0903 (0.019)	0.1376	163
	SB cons. 1	55	0.0665 (0.0026)	0.943 (0.016)	0.0772 (0.020)	0.0948	86
	SB cons. 2	55	0.0701 (0.0032)	0.955 (0.014)	0.0893 (0.023)	0.1147	104
	SB cons. 3	55	0.0625 (0.0195)	0.886 (0.025)	0.0739 (0.020)	0.0893	81
	SB cons. mean (sample 1–3)	55	0.0664	0.928	0.0801	0.0996	90.3
	SB cons. SE (sample 1–3)	55	0.00219	0.0213	0.00469	0.00770	70.0
	SB subsampling mean (5 × 11)	11	0.0663 (0.011)	0.910 (0.011)	0.0625 (0.022)	0.0706	43.8

Note

Measure of MHC II DQB diversity for all samples and subsamples for Shark Bay (SB) and Bunbury (BB). BB max. and SB max are based on the maximum number of samples for which we obtained MHC sequences. BB cons. is based on the samples for which we also have microsatellite data. SB samples 1–3 are based on subsamples that include the same number of calves, juveniles, adults, and females as in the BB cons. set of samples and for which we also have microsatellite data. SB cons. The final two rows show the mean values and standard errors of the mean, respectively, across all three subsamples (SB samples 1–3). Tabulated are *n* = the sample size; *π* = nucleotide diversity; *Hd* = haplotype diversity; *Ө_W_* = Watterson mutation estimator; *Ө_Eta_* = the mutation parameter theta based on number of mutations, *Eta*. Standard deviations for ***π***,* Hd*, and* Ө_W_* are shown in parentheses.

### 3.4 Microsatellite diversity

Neither population showed evidence for scoring errors due to stuttering or large‐allele dropouts for any of the microsatellite loci. We also did not detect evidence for null alleles for any of the loci. Linkage disequilibrium tests with Genepop showed that, among all comparisons of pairs of microsatellite loci, one pair appeared linked (Tur4_105 & MK8), but this linkage was only observed for BB, so it was unlikely to be a result of physical linkage. Departures from HWE expectations were observed for two microsatellite loci, Tur4_98 (SB) and KWM12 (SB & BB), after Bonferroni correction. Those two loci were removed from subsequent analysis. Consequently, all subsequent results are based on 23 loci.

**Figure 4:**
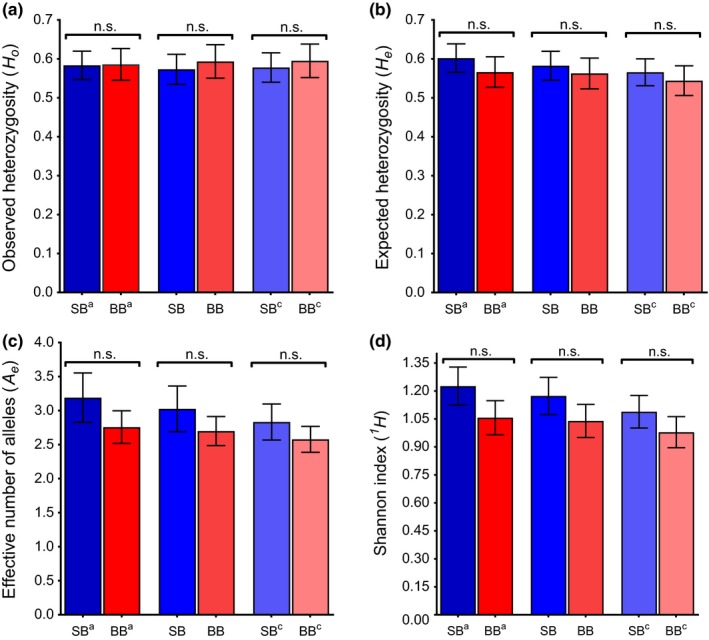
Microsatellite diversity (23 polymorphic loci), of dolphins in Shark Bay (SB; blue bars) and Bunbury (BB; red bars): (a) observed heterozygosity (*H_O_*), (b) expected heterozygosity (*H_E_*), (c) effective number of alleles (*A_e_*), and (d) Shannon index (*^1^H*). Each pairing of SB‐BB bars represents the results on the basis of each of the three sampling approaches: maximum sampling (SB^a^, BB^a^), conservative sampling (SB^b^, BB^b^), and subsampling (SB^c^, BB^c^). Whiskers depict the standard errors of the mean across the 23 loci. Significant values based on paired *t* tests: n.s. = nonsignificant (*p* > 0.05)

In contrast to the MHC results, microsatellite diversity showed no significant differences between SB and BB. However, there was a nonsignificant trend of SB being genetically more diverse than BB with respect to *H_o_*, *H_e_*, *A_e_*, and *^1^H* (Figure 4a–d; Tables 4 and 5). The conservative sampling approach showed average numbers of alleles per microsatellite locus of 5.68 in SB and 4.30 in BB; *H_o_* of 0.546 in SB and 0.588 in BB (*t* = 0.6482, *df* = 22; *p* = 0.5236; Figure 4a; Tables 4 and 5); *H_e_* of 0.578 in SB and 0.559 in BB (*t* = 0.5508, *df* = 22, *p* = 0.5873; Figure 4b; Tables 3 and 5); *A_e_* per locus of 2.98 (*SE* = 0.35) for SB and 2.70 (*SE* = 0.23) for BB (*t* = 1.011, *df* = 22, *p* = 0.3231; Figure 4c; Tables 4 and 5); *^1^H* of 1.17 in SB and 1.04 in BB (*t* = 1.752, *df* = 22, *p* = 0.0938; Figure 4d; Tables 4 and 5). There was also no significant difference between the SB subsamples with respect to the microsatellite measures of genetic diversity (Dryad/Table S5). Results of paired *t* tests for all sampling approaches are shown in Table 5.

**Table 4:** Microsatellite diversity measures

Pop	Sampling approach	*n*	*H_o_*	*H_e_*	*A_e_*	*^1^H*
Bunbury	BB max.	84	0.59^a^ (0.041)^a^	0.57^a^ (0.039)^a^	2.76^a^ (0.24)^a^	1.058^a^ (0.09)^a^
	BB cons.	55	0.588 (0.0465)	0.559 (0.0424)	2.696 (0.228)	1.037 (0.0938)
	BB subsampling mean (5 × 11)	11	0.590 (0.0465)	0.540 (0.0410)	2.577 (0.204)	0.978 (0.0884)
Shark Bay	SB max.	676	0.579 (0.0378)	0.598 (0.0383)	3.149 (0.379)	1.220 (0.104)
	SB cons. 1	55	0.551 (0.0422)	0.561 (0.0414)	2.886 (0.347)	1.135 (0.105)
	SB cons. 2	55	0.566 (0.0425)	0.587 (0.0382)	3.027 (0.352)	1.184 (0.101)
	SB cons. 3	55	0.576 (0.0404)	0.585 (0.0389)	3.026 (0.354)	1.185 (0.104)
	SB cons. Mean (SB cons. 1–3)	55	0.564 (0.0402)	0.578 (0.0388)	2.980 (0.348)	1.168 (0.102)
	SB cons. SE (SB cons. 1–3)	55	0.0073	0.0084	0.047	0.017
	SB subsampling mean (19 × 11)	11	0.572 (0.0393)	0.561 (0.0361)	2.800 (0.275)	1.083 (0.0895)

Note

Measure of microsatellite diversity based on the three sampling approaches (maximum, conservative and subsampling) for Shark Bay (SB) and Bunbury (BB). BB max.* and SB max. are based on the maximum number of samples for which we obtained microsatellite genotypes. BB cons. are based on the samples for which we also have MHC II DQB data. SB cons. samples 1–3 are based on the conservative sampling that include the same number of calves, juveniles, adults, and females as in the BB cons. set of samples and for which we also have microsatellite data. SB cons. The rows “SB cons. Mean” and “SB cons. SE” show the mean values and standard errors of the mean, respectively, across the three conservative SB subsamples (SB samples 1–3). The rows “subsampling mean” show the mean values based on the subsampling approach. Tabulated are *n* = the sample size; *H_o_* = observed heterozygosity; *H_e_* = expected heterozygosity based on Hardy–Weinberg expectations; *A_e_* = the number of effective alleles; *^1^H* = Shannon index value. Standard errors across the microsatellites are shown in parentheses.

a Microsatellite diversity measures based on the BB maximum sampling approach are from Manlik et al. (2018).

**Table 5:** Results of *t* tests comparing genetic diversity measures between Shark Bay and Bunbury for MHC and microsatellites based on the various sampling approaches

MHC subsampling	*t*	*df*	*p*
*π*	4.303	22	**0.0003**
*Hd*	0.191	22	0.8503
*Ө_W_*	2.299	22	**0.0314**
*Ө_Eta_*	2.234	22	**0.0359**

msat maximum sampling	*t*	*df*	*p*
*H_o_*	0.1364	22	0.8928
*H_e_*	1.015	22	0.3213
*A_e_*	1.182	22	0.2498
*^1^H*	2.073	22	0.0501

msat conservative sampling	*t*	*df*	*p*
*H_o_*	0.6482	22	0.5236
*H_e_*	0.5485	22	0.5882
*A_e_*	1.011	22	0.3231
*^1^H*	1.752	22	0.0938

msat subsampling	*t*	*df*	*p*
*H_o_*	0.4743	22	0.6400
*H_e_*	0.6572	22	0.5179
*A_e_*	1.003	22	0.3268
*^1^H*	1.529	22	0.1406

Note

Results for microsatellites (msat) are based on paired *t* tests comparing diversity values across 23 loci. Significant *p*‐values (*p* < 0.05) are shown in bold.

## 4 DISCUSSION

Compared to selectively neutral genetic variation, variation of adaptive genes, such as those of the MHC, is a better proxy for genetic diversity relevant to population viability (Oliver & Piertney, 2012; Sommer, 2005; Ujvari & Belov, 2011). A loss of adaptive genetic diversity reduces reproductive success and survival in the short‐term and ultimately diminishes the evolutionary potential of populations to adapt to environmental changes (Frankham, 2005; Frankham et al., 2010). Our results show that the more stable SB population, which displayed greater reproductive success (Manlik et al., 2016), harbors greater MHC diversity compared with the BB population that was forecast to decline. It is important to note that this was the case, regardless of the sampling approach. Our finding that microsatellites do not show any significant differences between SB and BB suggests that the higher MHC diversity in SB is unlikely due to differences in population size, because the resultant genetic drift is expected to affect MHC and microsatellites equally (although see Eimes et al., 2011). Therefore, it seems likely that other interactions, such as differential fitness or parasite pressure, are driving the observed MHC pattern.

The number of sequence variants we detected in both populations is unusually high, but a high number of single‐locus MHC class II variants have been detected in other cetacean populations as well (e.g. Xu et al., 2012). As mentioned in the Methods, we refrain from classifying the sequence variants as novel MHC alleles, but having followed the same methodology of inferring sequence variants for both populations allowed us to compare MHC sequence variation between the two populations. Further confirmation of alleles could be achieved by sequencing clones (Marsh et al., 2010) or by re‐genotyping all individuals with rare haplotypes (Ahmad et al., 2002).

### 4.1 Potential factors contributing to the inter‐population differences in MHC diversity

Differences in MHC diversity between the two populations might be related to fitness. Adult females in SB displayed higher reproductive success than BB females (Manlik et al., 2016), and preliminary data suggest that SB females with greater reproductive success also exhibit greater MHC DQB diversity than females with low reproductive success (Manlik, 2016). Another selective effect associated with the inter‐population difference in MHC diversity is differences in pathogen communities. High levels of MHC diversity can be maintained by balancing selection due to MHC's function in binding to pathogen‐derived antigens (Eizaguirre, Lenz, Kalbe, & Milinski, 2012; Takahata & Nei, 1990; Wegner, Reusch, & Kalbe, 2003). The signal that we detected by the *d_N_*/*d_S_* analyses relates to long periods, with time for mutations to accumulate very slowly, at a rate of about 10^−9^ per generation per nucleotide site. These patterns were originally proposed for differentiation between species, but the same patterns are expected for variation within a single population, though weaker (Kryazhimskiy & Plotkin, 2008). The higher ratios of nonsynonymous to synonymous substitutions that we observed in the MHC DQB region of both populations are consistent with balancing selection (Kimura, 1977; Yang & Bielawski, 2000). There are studies on numerous vertebrate taxa that show an association between pathogen load, infectivity, and MHC diversity (e.g., Paterson, Wilson, & Pemberton,^1998^; Sepil, Lachish, Hink, et al., 2013; Wegner et al., 2008). Vassilakos et al. (2009) proposed that differential pathogen pressure across the range of cetacean populations could explain geographic variation in MHC diversity.

Although the *d_N_*/*d_S_* analyses can detect balancing selection over long periods, on shorter time scales, there might be other influences, such as bottlenecks, or directional selection due to a recent change in pathogen load; these can be detected by Tajima's *D*, with the proviso that because it is sensitive to demographic and selective effects, they could cancel each other out. Bottlenose dolphin mortalities due to pathogens, such as the cetacean morbillivirus, have been reported in Western Australia (Stephens et al., 2014), and outbreaks are associated with high mortality (van Bressem et al., 2014; Di Guardo & Mazzariol, 2014). If this mortality is selective, then it could give a signal with Tajima's *D*, unless counteracted by some demographic effect. However, little is known about pathogen communities across geographic locations, including the two sites of this study. Other factors are unlikely to explain the differences in MHC diversity between SB and BB: Age and sex are unlikely because our sample sizes had equal numbers of each age class and sex; effects of mate choice (Kamiya, O'Dwyer, Westerdahl, Senior, & Nakagawa, 2014; Yamazaki & Beauchamp, 2007) are possible but unlikely because both SB and BB exhibit a promiscuous mating system (Connor, Richards, Smolker, & Mann, 1996; Smith et al., 2016). Regardless, the difference in MHC diversity between the two populations likely also confers a differential potential to respond to pathogen pressure.

The diverse function and variability of MHC genes reflect evolutionary adaptive processes and thus make them suitable candidates to evaluate genetic diversity relevant to conservation. In this study, we compared MHC genetic diversity and microsatellite diversity of two contrasting bottlenose dolphin populations. We revealed signals of selective processes acting on the MHC DQB in both populations. In comparison with the BB population, the more stable SB population exhibited larger MHC diversity. This is congruent with our hypothesis that the difference in reproductive output and viability between the two populations (Manlik et al., 2016) may be better reflected by adaptive genes of the MHC than putatively neutral microsatellite loci. However, it is important to point out that it is not possible to make conclusive population‐level inferences because we compared only two populations. Our results do not allow us to differentiate between cause and effect: Low MHC diversity could be driving population decline, and population decline could be diminishing MHC diversity. Those two explanations are not mutually exclusive. Both explanations would warrant monitoring MHC diversity of wild animal populations, either as an indicator (reflecting population declines) or as potential driver of population viability (causing population declines). In either case, the greater inter‐population difference in MHC diversity relative to microsatellite diversity adds to the growing body of evidence pointing to MHC diversity as a suitable marker for the conservation of vertebrates. Our results also suggest that the SB population, harboring larger MHC diversity, might have a greater potential to respond to a larger variety of pathogens, which would make it more resilient to environmental change.

## CONFLICT OF INTERESTS

None declared.

## AUTHORS' CONTRIBUTIONS

O.M., W.B.S., M.K., J.M. C.F., L.B., and R.C.C. devised this study. O.M. performed all MHC sequencing and analyses, genotyped microsatellite loci, assessed microsatellite diversity, performed molecular sexing analyses, and wrote the original draft. M.K., A.M.K., and S.J.A. collected most of the biopsy samples. M.K. and A.M.K. extracted DNA, genotyped microsatellite loci, and performed molecular sexing analyses. L.B. and S.J.A. contributed data (identity, sex, age, etc.) on the Bunbury dolphins. J.M., R.C.C., C.F., M.K., A.M.K., and S.J.A. contributed data (identity, sex, age, etc.) on the Shark Bay dolphins. W.B.S. supervised O.M. on this project and contributed to the genetic analyses. All authors reviewed and edited the manuscript.

## Data Availability

MHC DNA sequence alignment available in Dryad/Figure S1 (Manlik et al., 2019; https://doi.org/10.5061/dryad.73k278d). Microsatellite data: previously uploaded in supplement of published article (Manlik et al., 2018): https://doi.org/10.1111/mms.12555. Additional microsatellite data now in Dryad/Table S1, available from the Dryad Digital Repository: Manlik et al. (2019) https://doi.org/10.5061/dryad.73k278d. Various other data available in Dryad/Appendix (https://doi.org/10.5061/dryad.73k278d; Manlik et al., 2019), including: conservative sampling design and numbers (Table S2), MHC and microsatellite diversity measures, including *F_ST_* (Table S3), BLASTN results (Table S4), diversity measures and statistics for microsatellite subsamples (Table S5).
