# Supplementary material for: Is MHC diversity a better marker for conservation than neutral genetic diversity? A case study of two contrasting dolphin populations
Source: Ecol Evol. 2019 May 23;9(12):6986–98. doi: 10.1002/ece3.5265 (PMC6662329; doi:10.1002/ece3.5265)
Supplement: Supplementary file 1 [file ECE3-9-6986-s001.pdf]

Appendix/Dryad

Is MHC diversity a better marker for conservation than neutral genetic diversity? A case study of two contrasting dolphin populations

Authors:

Oliver Manlik, Michael Krützen, Anna M. Kopps, Janet Mann, Lars Bejder, Simon J. Allen, Celine Frère, Richard C. Connor and William B. Sherwin

```
Variant:  1           10                  20                  30                  40                  50                  60                  70                  79
Tu-DQB-1  C ACG GAG CGG GTG CGG GTC GTG GAC AGA TCC ATC TAT AAC CGG GAG GAG TTA GTG CGC TTC GAC AGC GAC GTG GGC GAG
Tu-DQB-2  • ••• ••• ••• ••• ••• ••• ••• ••• ••• ••• ••• ••• ••• ••• ••• ••• ••G ••• ••• ••• ••• ••• ••• ••• ••• •••
Tu-DQB-3  • ••• ••• ••• ••• ••• •A• ••• A•• ••• •A• ••• ••• ••• ••• ••• ••• •A• ••• ••• ••• ••• ••• ••• ••• ••• •••
Tu-DQB-4  • ••• ••• ••• ••• ••• ••• A•• A•• ••• •A• ••• ••• ••• ••• ••• ••• •A• A•• ••• ••• ••• ••• ••• ••• ••• •••

Variant:  80          90                  100                 110                 120                 130                 140                 150         157
Tu-DQB-1  TTC CGG GCG GTG ACC GAG CTG GGC CGG CCG GAC GCC GAG TAC TGG AAC AGC CAG AAG GAC ATC CTG GAG CGG AAA CGG
Tu-DQB-2  ••• ••• ••C ••• ••• ••• ••• ••• ••• TG• AT• ••• ••• ••• ••• ••• ••• ••• ••• ••• T•• ••• ••• ••• ••• •••
Tu-DQB-3  ••• ••• ••• ••• ••• ••• ••• ••• ••• ••• ••• ••• ••• ••• •TC ••• ••• ••• ••• ••• ••• ••• ••• GA• G•• •••
Tu-DQB-4  ••• ••• ••• ••• ••• ••• ••• ••• ••• ••• ••• ••• ••• ••• •TC ••• ••• ••• ••• ••• ••• ••• ••• •A• G•• •••

Variant:    160         172
Tu-DQB-1  GCC GAG CTG GAC ACG
Tu-DQB-2  ••• ••• ••• ••• •••
Tu-DQB-3  ••• ••C G•• ••• •G•
Tu-DQB-4  ••• •C• G•• ••• •••
```

Fig. A1 Alignment of MHC II DQB sequence variants detected as homozygous. Numbers above sequence alignment (first row) indicate the number of basepairs (i.e. position). The most prevalent sequence variant (Tu-DQB-1), which was found homozygous in individuals of the two *Tursiops* populations is listed first as reference. A dot (•) indicates an identical basepair with reference to Tu-DQB-1. Following Heimeier *et al*., 2009, MHC II DQB codons that encode peptide binding sites are indicated according to: Murray *et al*., 1995; Hoelzel *et al*., 1999; Seddon & Ellegren, 2002; Hayashi *et al*., 2003 and Baker *et al*., 2006. The peptide binding sites are highlighted in turquoise color. Primer sequences are not included in this alignment or the analysis.

Table A1 Microsatellite loci and primer sequences for Shark Bay and Bunbury population

Locus	Primer sequences		Multiplex	Label	Allele size range (bp)	No. of Alleles	References
	Forward (5'-3')	Reverse (5'-3')					
D22	ACATTCCCCAATAAAAGTTAAAGT	CTCTTTGACATGCCCTCACC	2	NED	110-124	5	(a)
KWM12*	CTGGGCACTGTCCTCTGAACATC	AGGAACGGCACATAAAGCACTGA	3	VIC	156-190	15	(b)
MK3	TGCATTCATGTAAAGGTGCG	CTGCAACTAGAGAAAGCC CG	3	NED	147-171	10	(c)
MK5	CTCAGAGGGAAATGAGGCTG	TGTCTAGAGGTCAAAGCCTTCC	3	6-FAM	205-219	6	(c)
MK6	GTCCTCTTTCCAGGTGTAGCC	GCCCACTAAGTATGTTGCAGC	1	PET	152-190	16	(c)
MK8	TCCTGGAGCATCTTATAGTGGC	CTCTTTGACATGCCCTCACC	3	PET	87-117	11	(c)
MK9	CATAACAAAGTGGGATGACTCC	TTATCCTGTTGGCTGCAGTG	3	6-FAM	168-178	6	(c)
Tur4_E12	CTGGGCACTGTCCTCTGAACATC	AGGAACGGCACATAAAGCACTGA	1	PET	256-280	6	(d)
Tur4_F10	TCTTGATGGCTCAGAGGATGATTTTAC	AGCCAAACTGAAGATGCAACTGACTAC	2	6-FAM	374-398	7	(d)
Tur4_66	GAGGGTGGAATGGGGACAAAAAT	TGAAGCCAGGAGACTAGGACAGGTT	1	VIC	185-205	6	(d)
Tur4_80	AGCCAATGTCAGGGTGCTGGAT	GGGGCTTCTTGGCCTCTGTAA	3	NED	287-331	9	(d)
Tur4_87	CCCCATATGATGCCTTTGTAAGTCC	AATTCCTTGTAACAAACCTCTTTATCT	2	VIC	178-194	5	(d)
Tur4_91	GTTGGCTCTCCAGCTCTCAGGT	CAGTGGCTCCCATCTGTATTAGTCA	2	PET	207-235	8	(d)
Tur4_98*	GTCCCCAGAACTTAGCACACTGTC	CAACTGGGGTCCAAAGAAAGAAG	1	NED	192-196	2	(d)
Tur4_105	CCCCGGCCTGCTTACCTCTG	CCGCCCCCTCCCCAAGTC	1	6-FAM	367-403	9	(d)
Tur4_108	ACAGGGACCTGAGTGGGTGTAAG	CTTCCCTGGGTCTCTAGGCTACC	1	6-FAM	258-270	2	(d)
Tur4_111	CTCTGTAAGCACCCGTCCTGTGTA	TTCCCGCAGAATTCTGTGAACC	1	VIC	287-307	5	(d)
Tur4_117	TTGCAGTCAGCGTTTTCCAGAGA	GCCAGCCCATCCTTCAGATTTC	1	6-FAM	175-191	5	(d)
Tur4_128	ACGTGCGCATGTCTTTGTCTTAT	CTTTGGACGGGGAGTAGAACCTA	1	NED	295-311	5	(d)
Tur4_132	CTTCCATGCGCCAGACAACCT	TGGCAAGATGAGAGGGAAAGAGG	3	VIC	326-334	3	(d)
Tur4_138	GTGGCTTACCATGGTGGATTCAG	GCATGGCCATAAAGGGAGGAG	2	6-FAM	207-231	7	(d)
Tur4_141	CACAAGCCTCAACCCTGGTGT	CTAGTCTGCCAATCTGCCCTACAG	2	NED	218-286	13	(d)

Tur4_142	GGCCCCCTTTTCCATCCTCA	CCAGCCCCCAAAATCACGAGT	3	PET	330-346	5	(d)
Tur4_153	TGAGTAACCCCAATCTCGGTCTCT	CCAGCCCCCAAAATCACGAGT	3	PET	215-219	2	(d)
Tur4_162	GCCAACCTCCAGGCAAACACTC	TGCAGTCAACCTGAGGCAAGTCTC	3	6-FAM	365-419	6	(d)

Primer sequences for 25 microsatellite loci used for genotyping Shark Bay and Bunbury bottlenose dolphins. *Loci KWM12 and Tur4_98 were removed from final analysis because they departed from Hardy-Weinberg-Equilibrium expectations. Annealing temperature for polymerase chain reaction was 60 °C for all three multiplex PCR reactions. Fluorescent labels for forward primers are listed in the column “Labels”. The range of allele sizes and the total number of alleles (‘No. Alleles’) for each locus of all individuals sampled in Shark Bay and Bunbury are listed. References for the primer sequences are (a): Shinohara et al. 1997; (b): Hoelzel et al., 1998; (c): Krützen et al., 2001; (d): Nater et al., 2009. This table is modified from supplementary Table S1, Manlik et al. (2018): Manlik, O., Chabanne, D., Daniel, C., Bejder, L., Allen, S.J., Sherwin, W.B. 2018. Demography and genetics suggest reversal of dolphin source-sink dynamics, with implications for conservation. Mar. Mammal Sci., doi:10.1111/mms.12555.

Table A2 Conservative sampling of individuals with MHC and microsatellite data, which included equal numbers of males, females, calves (calf), juveniles (juv.) and adults (adu.) for each of the two populations. Age classes, i.e. calves, juveniles and adults are defined as in Manlik *et al*. (2016).

**Cons. Sampling**	**calf**	**juv.**	**adu.**	**males**	**females**	**Total**
BB cons.	2	15	38	32	23	55
SB sample 1	2	15	38	32	23	55
SB sample 2	2	15	38	32	23	55
SB sample 3	2	15	38	32	23	55

Table A3 Microsatellite and MHC II DQB diversity measures of the two sampling locations of Shark Bay. Measures of microsatellite diversity (A_e = effective number of alleles; ^1H = Shannon index; F_{IS} = fixation index) and MHC II DQB nucleotide diversity (π) are listed for the two sampling locations in Shark Bay: East Shark Bay (ESB) and West Shark Bay (WSB). Subpopulation fixation index (F_{ST}) indicates the degree of differentiation between ESB and WSB.

	Microsatellite diversity						MHC II DQB nucleotide diversity		
**Location**		***n***	A_e	^1H	F_{IS}	F_{ST}		***n***	π
**ESB**	**Mean**	409	3.131	1.205	0.030		**Mean**	231	0.06548
	**SE**		0.365	0.102	0.009		**SD**		0.00150
ESB vs WSB						0.006			
**WSB**	**Mean**	258	3.180	1.225	0.026		**Mean**	45	0.06197
	**SE**		0.334	0.099	0.010		**SD**		0.00213

Table A4 MHC II DQB variants* and BLASTN hits. Names of MHC II DQB sequence variants found in the Shark Bay and Bunbury *Tursiops aduncus* populations are listed in order from Tu-DQB-1 to Tu-DQB-3. Only the four sequence variants of thirty-nine individuals that are homozygous for all 172 nucleotide sites are listed here. Sequence identities to alleles that closely match these sequence variants and are published on GenBank are shown along with allele names and species in which they were found. Note that Tu-DQB-4 is identical (100% identity at 100% coverage) to the MHC II DQB exon 2 allele *Dede-a*, which was reported in the common dolphin (*Delphinus delphis*). Additional sequence variants, i.e. haplotypes, which were inferred by haplotype reconstruction, are not shown here (see main text).

**Variant**	**Identity**	**Allele**	**Species**
Tuad-DQB*1	99%	Tutr-DQB*27	*Tursiops truncatus*
	100%	Tutr-DQB*06 *(Note: 98% coverage)*	*Tursiops truncatus*
	98%	Stco-DQB*04	*Stenella coeruleoalba*
	98%	Orbr-a	*Orcaella brevirostris*
	97%	Cehe-DQB*02	*Cephalorhynchus hectori*
	97%	Tutr-DQB*25	*Tursiops truncatus*
Tuad-DQB*2	100%	Tutr-DQB*03 *(Note: 98% coverage)*	*Tursiops truncatus*
	98%	Stco-DQB*04	*Stenella coeruleoalba*
	98%	Cehe-DQB*02	*Cephalorhynchus hectori*
	97%	Tutr-DQB*01; Tutr-DQB*15	*Tursiops truncatus*
Tuad-DQB*3	99%	Stco-DQB*06	*Stenella coeruleoalba*
	99%	Tutr-DQB*24 (Note: 98% coverage)	*Tursiops truncatus*
	99%	Tutr-DQB*13 (Note: 98% coverage)	*Tursiops truncatus*
	98%	Stco-DQB*05	*Stenella coeruleoalba*
Tuad-DQB*4	100%	Dede-a	*Delphinus delphis*
	99%	Tutr-DQB*01	*Tursiops truncatus*
	98%	DQB*0101 (LOC101278540)	*Orcinus orca*
	98%	Tutr-DQB*02	*Tursiops truncatus*

Table A5 Comparison of microsatellite measures within three SB subsamples. Mean values for microsatellite observed heterozygosity (H_o), expected heterozygosity relative to Hardy-Weinberg Equilibrium expectations (H_e), effective number of alleles (A_e) and Shannon indices (^1H) are shown for each of the subsamples (SB sample 1-3). *F* and *p*-values for analysis of variance (ANOVA) test are tabulated comparing the three subsamples. *ANOVA test on A_e was based on log-transformed values because the original values were not normally distributed.

	**SB sample 1**	**SB sample 2**	**SB sample 3**	***F***	***p***	**Sig.**
H_o	0.551	0.566	0.576	0.0919	0.9124	ns
H_e	0.561	0.587	0.585	0.130	0.8793	ns
A_e	2.931	3.094	3.051	0.0788*	0.9243*	ns*
^1H	1.141	1.194	1.185	2.886	0.0655	ns
